# Impact of implementation of the WHO FCTC on the tobacco industry’s behaviour

**DOI:** 10.1136/tobaccocontrol-2018-054808

**Published:** 2019-01-18

**Authors:** Stella Aguinaga Bialous

**Keywords:** WHO FCTC, impact assessment, research design

The tobacco industry has a decades-long history of opposing any measure that attempts to regulate its product, its marketing, pricing or social acceptability.^1 2^ The entry into force of the WHO Framework Convention on Tobacco Control (FCTC) in 2005, and the approval, by FCTC parties, of the Guidelines for Implementation of Article 5.3 in 2008 (to protect tobacco control policies from the ‘commercial and other vested interests of the tobacco industry’), have not yet served to completely prevent the tobacco industry from interfering with tobacco control policies.^3^

FCTC parties adopted, at the sixth Conference of the Parties in 2014, decision FCTC/COP6(15)^4^ calling for an independent expert group to assess the impact of the WHO FCTC and to report their findings at the seventh Conference of the Parties (COP7) in 2016. The decision called for a report on tobacco industry responses to the FCTC as one of three reviews commissioned by the Convention Secretariat. This commentary draws from the report originally prepared for COP7,^1^ updated to reflect additional evidence of tobacco industry interference since.

## The tobacco industry’s response to the WHO FCTC

The report of the expert group assessing the impact of the FCTC noted that ‘aggressive action by the global tobacco industry to oppose tobacco control measures and to undermine Article 5.3’ remains an obstacle to the implementation of the FCTC.^5^

The tobacco industry has opposed the FCTC since its inception, proposing alternative, voluntary policies and frameworks, and influencing a few selected countries to intervene in their favour.^1^ One of the industry’s options, facing the inevitability of the FCTC, was to engage with government and promote itself as part of the solution. Once the FCTC was adopted in May 2003, and entered into force in 2005, the tobacco industry responded with efforts to avoid legislation, weaken legislation that was approved and/or interfere with implementation of tobacco control measures. These strategies were deployed globally, but low-income and middle-income countries  were particularly vulnerable.^2^

As parties ratified and implemented the Convention, the industry attempted to provide input into parties’ tobacco control measures arguing that it supported ‘reasonable’ regulations, and often entering into voluntary agreements and partnerships with governments. The regulations supported by the tobacco industry were not aligned with either the intent or the letter of the FCTC and its guidelines. Recently released documents indicate that the tobacco industry attempted to use bribery and intimidation to influence the outcomes of the FCTC discussions.^6 7^

## The tobacco industry’s response to the WHO FCTC Article 5.3

There is growing evidence of the industry’s response to Article 5.3 specifically. Shortly after the approval of the Article 5.3 Guidelines in 2008, Paul Adams, then British American Tobacco’s Chief Executive, stated

…[W]e fully agree that the manufacture, distribution and sale of tobacco products should be regulated. But these ’guidelines' raise serious questions about real best practice in policy making. They are a potential recipe to vilify and marginalise legitimate, tax-paying, regulated businesses, employing thousands of people, and risk forcing tobacco products ’underground' where the illicit, non-taxpaying, unregulated trade is already flourishing… despite the clamour for ’denormalisation,' exclusion and extremism being promoted by many anti- tobacco activists, many governments seek balanced regulation that is transparent, accountable, proportionate and properly targeted.^8^

Despite the tobacco industry’s interference, Article 5.3 has been playing an increasingly significant role in protecting the Convention and public health against the tobacco industry’s interests, raising awareness and creating mechanisms of industry monitoring that facilitate shining a light on industry’s behaviour.^5^ It is increasingly difficult for the tobacco industry to stay ‘behind the scenes’ or to hide behind front groups. Although these practices continue, there is a heightened sense of alertness and tobacco control advocates, and the media, are quicker to highlight the tobacco industry links to these front groups. In 2017, the UN Global Compact decided to no longer list tobacco companies as ‘socially responsible’.^9^ While this is progress, additional efforts are needed to stop the tobacco industry, and industry-funded groups, from participating in the work of intergovernmental organisations, bringing policy coherence between UN agencies and the FCTC.

The tobacco industry criticises its exclusion from the FCTC deliberations as lacking transparency and input from stakeholders, ironically, often using Article 5.3 as an argument, claiming that Article 5.3 guidelines do not ban interactions with the tobacco industry. During the eighth Conference of the Parties (COP8), in October 2018 in Geneva, Philip Morris International (PMI) was hosting a science event promoting its new products.^10^ Moira Gilchrist, PMI’s Scientific and Public Communications Officer, used PMI Science’s Twitter account to invite the Head of the Convention Secretariat for a visit. In the comments to her invitation, there was a question if Dr Gilchrist expected delegates to the COP8 to violate Article 5.3, to which Dr Gilchrist replied, “Of course. Art. 5.3 isn’t a barrier to scrutiny. It calls for transparency. We welcome anyone to meet with us transparently to verify our science and our actions.” (https://twitter.com/PMIScience/status/1046745863081668615) This misrepresents the Article 5.3 Guidelines which states that meetings with industry are called by governments for regulatory purposes only. Her comment was similar to interpretation previously found in PMI’s ‘Just the Facts’ website page titled ‘FCTC Article 5.3: Misinterpreted To The Extreme’.

And last, but not least, the tobacco industry continues to invest in its ‘corporate social responsibility’ or ‘sustainability’ programmes, globally, with what appears to be an increased emphasis on issues related to illicit trade and, lately, emphasising how the industry will participate in reaching the Sustainable Development Goals.^11^

## The tobacco industry’s behaviour as a consequence of WHO FCTC

Three areas of interference exemplify the tobacco industry’s response to the FCTC: efforts in promoting harm reduction, litigation, and increased use of international trade agreements as an argument to oppose tobacco control policy and the Protocol.

### Investing in ‘reduced harm’ tobacco products

A possible area of enhanced tobacco industry activity in response to the FCTC is its renewed efforts, and investments, in so-called ‘reduced harm’ tobacco products. While the search for a ‘reduced harm’ product is not recent, in the past few years there has been an increase in industry statements fostering their image as a stakeholder in tobacco control through the creation of allegedly less harmful products. These products would maintain markets open and available to the industry and avoid more strict tobacco control measures and the pursuit of an endgame. A recent example of these products is heated tobacco products (HTP) which emerging independent research demonstrates have no health benefits.^12 13^ At COP8, parties adopted a decision to treat, and regulate, HTPs as tobacco products under the FCTC Articles and guidelines.^14^

These ‘reduced harm’ strategies need to be seen in the context of tobacco industry’s long history of manipulating cigarette design and ingredients to increase its appeal and palatability, as well as its opposition to policy measures implementing of Articles 9 and 10 of the FCTC. For example, Brazil’s regulation banning additives has been legally disputed by the industry since it was approved in 2012. While in March 2018, the Supreme Court of Brazil found in favour of the ban, it did not dismiss lower court cases, thus the ban is, as of October 2018, still waiting for implementation. At the same time, the industry continues to oppose evidence-based harm reduction policies, that is, policies that have led to decrease in tobacco use prevalence and consumption such as smoke-free environments, tax increases, plain packaging, among other FCTC-supported tobacco control policies.

Further, PMI now funds the Foundation for a Smoke-Free World^12^ which purports to support harm reduction, and is using its vast wealth to propose a partnership with the WHO, although its support for the above-mentioned evidence-based articles of the FCTC remain to be seen.^15^ A partnership with this foundation would be in breach of Article 5.3 as the foundation is funded entirely by the tobacco industry.^16^

### Increased use of litigation to oppose implementation of the FCTC

On the issue of litigation, it is clear that the tobacco industry is using this strategy to block FCTC progress. A database of litigation maintained by the Campaign for Tobacco Free Kids^17^ includes 299 cases that were ‘direct challenges to government policies related to tobacco control/public health’. Such cases are globally distributed and affect high-income, middle-income and low-income parties. It is important to note that this is just a subset of the over 1000 cases documented, including litigation against implementation of Articles 13, 11 and 8 of the Convention.

### International trade agreements as an argument to oppose the FCTC

The industry has claimed that tobacco control measures are in breach of international trade agreements before (at least since 1992), and this argument remains a strategy in which the tobacco industry uses trade agreements, including bilateral or investors’ agreements, to oppose implementation of the FCTC. The internal tobacco industry documents dated from the time the FCTC was being discussed clearly outline the use of international trade as a potential mechanism for the tobacco industry to use against the FCTC.^18^ The claims that the FCTC implementation could be in breach of trade agreements is an argument used more intensely in the past 5 years, most notably in the cases of Philip Morris International against Uruguay’s cigarette pack regulations and the case against Australia’s plain pack regulations. In both cases, the industry lost their plea to have these regulations overturned on trade grounds. Nonetheless, trade arguments continue to be used in tobacco industry’s submissions, litigation or threat of litigation opposing implementation of pictorial warning labels and plain pack regulations in several countries, from Namibia, to Jamaica to Ireland and beyond. As more countries implement these packaging regulations, the chilling effect from these litigation threats could decrease.^18^

## The protocol to eliminate illicit trade in tobacco products

The tobacco industry has been, directly and indirectly, engaged in illicit trade of tobacco products for decades.^19^ Research^20^ demonstrates that the industry’s involvement is ongoing, despite its public relations efforts to claim that they are partners in addressing contraband. It is of note that less than 5% of the illicit trade market is counterfeited products,^21^ although this seems to be the focus of tobacco industry’s ‘partnership’ efforts.

The past decade has seen the industry intensify its efforts to interfere with the development and implementation of the protocol^19^ and aggressively promote its own track and tracing system^20^ which does not conform with the protocol standards. The entry into force of the protocol in September 2018, and the first Meeting of the Parties (MOP1) in October 2018, requires that parties remain alert to the industry’s efforts to promote itself as a partner in the protocol implementation. Transparency measures approved by MOP1 will support parties in ensuring that the protocol is implemented free of tobacco industry interference.^22^

There is evidence that the tobacco industry started to prepare a response to the Convention at approximately the same time that the discussion to develop the treaty started.^1 23 24^ The response of the tobacco industry to the Convention has not, in itself, changed from previous documented strategies used by the tobacco industry to oppose tobacco control; however, the intensity of some tactics (eg, litigation) has changed, and it appears that some new alliances and front groups were added to previously reported ones.

There is global progress in industry monitoring and sharing information about tobacco industry activities, a progress that will be accelerated with the launch of the tobacco industry observatories and the WHO FCTC Knowledge Hub on Article 5.3, in addition to the recently launched Bloomberg Philanthropies-funded Stopping Tobacco Organizations and Products (STOP) project. Information about the tobacco industry interference could inform parties’ efforts to use litigation in order to hold the tobacco industry accountable for delaying progress in implementation of the WHO FCTC.

There is also evidence that Article 5.3 is emerging as a significant and effective measure to halt the tobacco industry’s efforts to interfere with tobacco control and public health, and that the tobacco industry is making efforts to misrepresent Article 5.3 as it continues to claim that it should be involved in decision-making related to FCTC implementaion.  Parties need to remain alert and ensure that Article 5.3 is implemented across different government sectors, aiming at policy coherence that does not favour the interests of the tobacco industry over parties’ obligations under the FCTC.
